# Medical Statistics

**Published:** 1901-09-28

**Authors:** 


### MEDICAL STATISTICS.

At the end of his most interesting address as President of the Economic Section of the British Association, Sir Robert Giffin appeals to all to give more attention to common statistics and the ideas derived from them. It cannot fail to be of interest both to the medical profession and the public to have some idea of the relative causes of death now, as compared with a period say one hundred years ago, and thus to gain some idea of the diseases which have been stamped out by modern sanitation and those which have been engendered by modern modes of living. Unfortunately, the science of medicine does not permit of mathematical precision in its statistics, owing to the fact that diagnosis is continually being improved upon, so that many diseases which would now be tabulated under distinct headings would have formerly been thrown together in one class under some vague name.

In the "Annual Register" for 1800 we find a general table of births and deaths in which it is stated that 19,176 persons were christened and 23,068 buried, and underneath is given the chief causes of death. They are interesting as reflecting the medical knowledge of the time. Consumption ranks first with 5,721, and in order of frequence then follows convulsions, 4,512; fevers of all kinds, 2,712; smallpox, 2,409; aged, 1,742; dropsy, 1,003; asthma and phthisic (sic), 801; inflammation, 593; and various other less frequent diseases and accidents complete the list. Now, although little can be learnt from this list, two prominent facts at once claim attention. It is doubtful whether consumption did not include many other diseases which were not tuberculous, and the class "asthma and phthisic" seems to indicate that some endeavour was being made to separate phthisis of the lungs from other tuberculous diseases; but it is perfectly clear that smallpox was particularly well known, so that we can trust those figures absolutely. Convulsions probably included most of the diseases of childhood, and "fevers of all kinds," sufficiently indicates by its name the class of affections included. We may thus safely assume that smallpox was the second most frequent cause of death at the commencement of the nineteenth century, allowing that tuberculous diseases ranked first.

Now, in spite of all the medical discoveries of the last century, and the improvement in the general mode of sanitation, the single disease which has been removed from nearly the top to the bottom of the list is smallpox, and, as far as we can judge by the classification, the remainder of the diseases still practically maintain their relative order of deadliness. These simple figures contain a great lesson for all those who argue that the abolition of smallpox has been due simply to improved sanitation, for it is obvious that if such were the case many other of the specific infective diseases would have shared the same fate. But if no prophylactic can be for one moment compared to vaccination in its effects in ridding the community of a horrible and deadly scourge, yet isolation and disinfection have greatly reduced the mortality from the other infective diseases, and careful attention to sewage disposal and water supply has enormously diminished the incidence of enteric fever.

The general result of the improvement in medical knowledge and sanitary surroundings is best shown by the death-rate, and here we are on safe and certain ground, for no question of diagnosis arises. In 1851 the death-rate per 1,000 in England was 22 and that has steadily fallen with an occasional rise (only once above this level, when it reached 22·6 in 1871) until it reached the lowest rate for the last half of the century in 1899, when it stood at 18·3. Even this is high compared with the death-rate for Australasia, which from 16·75 from 1861-1865, has fallen to 12·39 from 1896-1899, but here we must make the allowance that the whole population is younger owing to the number of immigrants. To many the result of the recent census came as a surprise, for it was confidently anticipated that the increase in the population would be smaller than in the previous decennial period. Instead we find that the increase was 873,000 greater than that recorded in 1891, the rate in percentages being 12·17 in 1901 as against 11·65 in 1891. This result has been obtained in spite of the fact that the birth-rate has steadily fallen during the period until that of 1899, was the lowest on record. The explanation, of course, is that the death-rate has also fallen to such an extent as to more than counterbalance the effect of the diminished number of births. All this speaks well for the healthiness of the country in spite of the exodus from the open fields into densely populated streets and factories. This brings us to the effects of occupation on mortality, and here some startling facts are told by the figures. Coalminers are less liable to phthisis than any other class of workers with the exception of farmers and fishermen. This disease, on the other hand, causes a high mortality among tailors, printers, wool and cotton workers, potters, and costermongers. The highest mortality figure of all is found in inn servants and others addicted to intemperate habits.

